# Cyberbullying and substance use in Brazilian adolescents: associations with depression, emotional regulation, and emotional vulnerability

**DOI:** 10.1192/j.eurpsy.2026.10845

**Published:** 2026-07-31

**Authors:** A. L. Monezi Andrade, E. M. Freires, L. S. da Silva, C. Romualdo, W. A. de Oliveira

**Affiliations:** School of Life Sciences, Pontifical Catholic University of Campinas, Campinas, Brazil

## Abstract

**Introduction:**

Adolescence is a time of emotional vulnerability and exposure to risky behaviors. A growing body of evidence indicates that cyberbullying-related actions may be linked to substance use, symptoms of depression, anxiety, and stress, and may impair emotional regulation. This study explored these connections in Brazilian adolescents, aiming to understand the combined effect of substance use and cyberbullying on mental health.

**Objectives:**

We investigated a potential association between substance use and cyberbullying (victimization and aggression) in adolescents and compared emotional vulnerability levels among those identified as at risk or not at risk for substance use.

**Methods:**

This is an exploratory, cross-sectional study involving a sample of 321 adolescents (aged 13–17). Risk for substance use was assessed using the CRAFFT/CESARE instrument. The instruments evaluated emotional symptoms (DASS-21), difficulties in emotion regulation (DERS-18), involvement in cyberbullying (Florence Scales), and quality of life (KIDSCREEN-10), along with measures of digital use (IAT, SPAI-BR, SAS-SV, STDS). Analyses included chi-square tests and ANOVA for between-group comparisons. Additionally, a network analysis (NA) was conducted to identify the main variables associated with substance use and cyberbullying. In all analyses, a significance level of 5% was adopted.

**Results:**

About 24% of adolescents were classified as at risk for substance use. Compared to those not at risk, these adolescents reported higher levels of depression, anxiety, and stress, more difficulties with emotion regulation, and increased involvement in cybervictimization and aggression. Additionally, they showed more problematic use of digital technologies, especially smartphones and social media. There were no significant differences in quality of life. Network analysis indicated that alcohol use was the most central variable, directly linked to age, cyberbullying victimization, and emotional regulation difficulties. Impulsivity also proved to be central, being associated with both substance use and cyberbullying.

**Conclusions:**

The data showed that substance use among adolescents was linked to increased emotional vulnerability and higher involvement in cyberbullying, both as victims and perpetrators. Impulsivity and alcohol use, in particular, stood out as key factors in this model.

**Disclosure of Interest:**

None Declared

